# Quantitative CT‐derived vessel metrics in idiopathic pulmonary fibrosis: A structure–function study

**DOI:** 10.1111/resp.13485

**Published:** 2019-02-20

**Authors:** Joseph Jacob, Michael Pienn, Christian Payer, Martin Urschler, Maria Kokosi, Anand Devaraj, Athol U. Wells, Horst Olschewski

**Affiliations:** ^1^ Department of Respiratory Medicine University College London London UK; ^2^ Centre for Medical Image Computing University College London London UK; ^3^ Ludwig Boltzmann Institute for Lung Vascular Research Graz Austria; ^4^ Institute of Computer Graphics and Vision Graz University of Technology Graz Austria; ^5^ Ludwig Boltzmann Institute for Clinical‐Forensic Imaging Graz Austria; ^6^ Interstitial Lung Disease Unit Royal Brompton Hospital London UK; ^7^ Department of Radiology Royal Brompton Hospital London UK; ^8^ Division of Pulmonology, Department of Internal Medicine Medical University of Graz Graz Austria

**Keywords:** interstitial lung disease, lung fibrosis, radiology and other imaging, respiratory structure and function

## Abstract

**Background and objective:**

This study aimed to investigate whether quantitative lung vessel morphology determined by a new fully automated algorithm is associated with functional indices in idiopathic pulmonary fibrosis (IPF).

**Methods:**

A total of 152 IPF patients had vessel volume, density, tortuosity and heterogeneity quantified from computed tomography (CT) images by a fully automated algorithm. Separate quantitation of vessel metrics in pulmonary arteries and veins was performed in 106 patients. Results were evaluated against readouts from lung function tests.

**Results:**

Normalized vessel volume expressed as a percentage of total lung volume was moderately correlated with functional indices on univariable linear regression analysis: forced vital capacity (R^2^ = 0.27, *P* < 1 × 10^−6^), diffusion capacity for carbon monoxide (DL_CO_; R^2^ = 0.12, *P* = 3 × 10^−5^), total lung capacity (TLC; R^2^ = 0.45, *P* < 1 × 10^−6^) and composite physiologic index (CPI; R^2^ = 0.28, *P* < 1 × 10^−6^). Normalized vessel volume was correlated with vessel density but not with vessel heterogeneity. Quantitatively derived vessel metrics (and artery and vein subdivision scores) were not significantly linked with the transfer factor for carbon monoxide (K_CO_), and only weakly with DL_CO_.

On multivariable linear regression analysis, normalized vessel volume and vessel heterogeneity were independently linked with DL_CO_, TLC and CPI indicating that they capture different aspects of lung damage. Artery–vein separation provided no additional information beyond that captured in the whole vasculature.

**Conclusion:**

Our study confirms previous observations of links between vessel volume and functional measures of disease severity in IPF using a new vessel quantitation tool. Additionally, the new tool shows independent linkages of normalized vessel volume and vessel heterogeneity with functional indices. Quantitative vessel metrics do not appear to reflect vasculopathic damage in IPF.

## INTRODUCTION

Idiopathic pulmonary fibrosis (IPF) is a progressive fibrosing lung disease associated with a median survival from diagnosis of 3–5 years.^1^, ^2^ Pulmonary function tests (PFT) including forced vital capacity (FVC)^2^ and diffusion capacity for carbon monoxide (DL_CO_)^3^ as well as visual analysis of parenchymal features on computed tomography (CT) images, including the extent of interstitial lung disease (ILD)^4^ and honeycombing,^1^, ^5^ form the basis of determining baseline disease severity in IPF.

Recent advances in computer technology have resulted in the development of tools capable of classifying and quantifying parenchymal features on CT data sets.^6^, ^7^ Whilst the majority of automated parenchymal features reflect patterns scored visually by radiologists, there has been increasing focus on novel CT patterns that computer tools can recognize, but which cannot be quantified visually. An example is the vessel‐related structures (VRS) readout determined by CALIPER,^7^ which was able to powerfully predict outcome in patients with IPF.^8^, ^9^ However, until now, CALIPER has been the only tool used to evaluate vasculature in patients with lung fibrosis.

Our study therefore aimed to use a new vessel quantitation tool to determine the volume of pulmonary vessels in patients with IPF. Extra segmentation features of the computer tool allowed pulmonary arteries and veins to be distinguished as well as new vessel parameters including vessel density, tortuosity and heterogeneity to be quantified. Computer‐derived vessel metrics were evaluated against PFT in a structure–function analysis.

## METHODS

### Study design

IPF patients presenting to the Royal Brompton Hospital and diagnosed by a multidisciplinary team using established guidelines^10^ were retrospectively identified. Patients for whom a complete non‐contrast volumetric CT scan was available were included in the study population. PFTs were considered if obtained within 3 months of the CT scan and included forced expiratory volume in the first second (FEV_1_), FVC, total lung capacity (TLC), DL_CO_, transfer factor for carbon monoxide (K_CO_) and the composite physiologic index (CPI).^11^ CT and pulmonary function protocols are included in Appendix S1 (Supplementary Information). Approval for this study of clinically indicated CT and pulmonary function data with a waiver for consent was obtained from the Institutional Ethics Committee of the Royal Brompton Hospital.

### Computer analysis of CT imaging

Image pre‐processing with a 1‐voxel wide median filter and segmentation of lung and airways was performed on the whole CT images using the Chest Imaging Platform.^12^ The vessel segmentation was performed with in‐house developed software. A detailed description and validation of the automatic vessel extraction algorithm is presented by Payer *et al*.^13^ Briefly, a multi‐scale vessel enhancement filter produces images with a high response for tubular structures as well as the respective radius and an estimate for the tube orientation. Optimized vessel paths with sub‐voxel accuracy are generated from regularly spaced maxima of the vesselness response following the tubular structures. The vessel trees are reconstructed from these paths and subsequently separated at the bifurcations into individual vessel segments. Only segments with diameters between 2 and 10 mm are included. Finally, arteries and veins are labelled by exploiting that arteries and veins are roughly uniformly distributed in the lung and that bronchi run approximately parallel and in close proximity to the arteries. The algorithm results in properly labelled and morphologically characterized vessel segments in most subjects.^13^

### Validation of vessel segmentations

Overlay colour maps of the vessels captured by the computer algorithm were superimposed on the CT images and were visually assessed by a radiologist (J.J.) to check the adequacy of pulmonary vessel extraction. Quality control of vessel extraction was evaluated using two metrics scored to the nearest 5%: (i) proportion of labelled structures that were not vascular in origin and (ii) proportion of vessels that were not labelled at all by the computer tool. For both metrics, a threshold of >10% error was used to exclude subjects. In addition, pulmonary artery/vein separation was evaluated by the same radiologist and subjects where >20% of the vessels were mislabelled as arteries or veins were excluded from any artery or vein sub‐analysis.

### Calculation of morphological readouts

Readouts were analysed for the whole lung (left and right lungs combined) and individually for three zones of equal volume (upper zones, middle zones and lower‐zones). The normalized vessel volumes for arteries, veins and all vessels were calculated by normalizing the cumulative volumes of the segmented arteries, veins and the combined vessel trees, respectively, to the subject's quantitatively derived total lung volume (for total lung scores) and zonal volume (for zonal scores). The number of vessel segments was also normalized to the respective subject's total/zonal lung volume calculating the vessel density. The tortuosity of the vessel segments was determined using the distance metric.^14^, ^15^ This is calculated as the ratio between the length of a vessel segment along its centre line and the Euclidean distance between its end points. The median distance metric was used as measure of vessel tortuosity. The distribution width between the 15th and the 85th percentile of distance metric values was considered as measure for vessel heterogeneity. Additionally, the mean lung attenuation was calculated as the average X‐ray attenuation of the lung parenchyma (lung segmentation after extraction of vessels and airways).

### Statistical analysis

Statistical analyses were performed with SPSS (IBM SPSS Statistics for Macintosh, Version 20.0 (IBM Corp., Armonk, NY, USA). Data are given as medians with ranges, means with SD or numbers of patients as appropriate. Group differences were examined using Student's t‐test for continuous variables, chi‐square test for categorical variables and the Mann–Whitney U‐test for non‐normally distributed median values. Univariable and multivariable linear regression analyses were performed to explore relationships amongst various quantitative CT metrics and between CT metrics and PFT.

## RESULTS

### Baseline data

**Figure 1:**
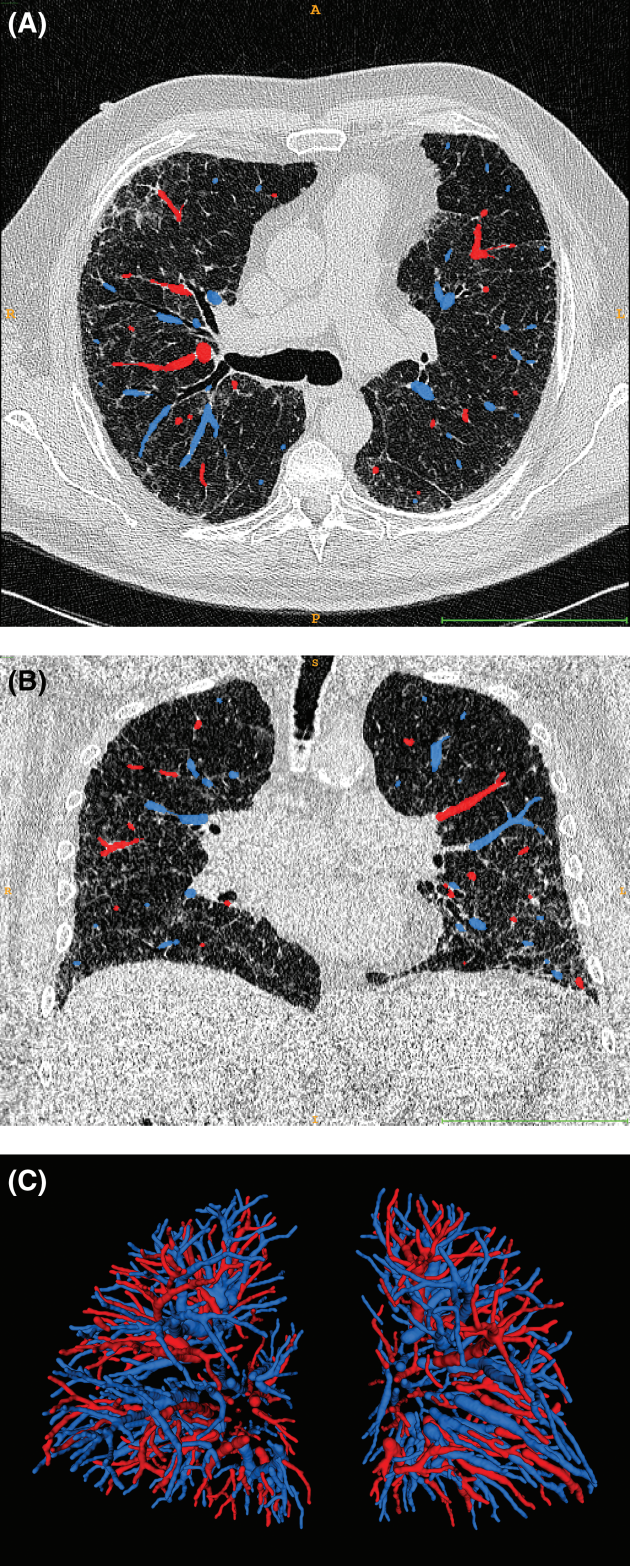
Transversal (A) and coronal (B) thoracic computed tomography (CT) images of a representative patient with overlays of the arteries (blue) and veins (red) and 3D rendering of the arterial and venous vessel trees in this patient (C). The bar at the bottom of (A) and (B) is 10 cm wide.

The study population comprised 176 IPF patients. Of the 176 CT, 4 (2%) could not be segmented due to motion artefacts. Twenty further subjects were excluded for either having >10% of the lung vessels not segmented or >10% of structures labelled as vessels representing non‐vascular (often coarse fibrotic) artefact. The 24 excluded patients had more severe baseline disease than the 152 patients included in the study (Table S1, Supplementary Information). Patients with >20% artery/vein misclassification (*n* = 46) were excluded from all artery and vein analyses. Demographic and baseline functional and quantitative results for the 152 study subjects are shown in Table 1. Representative CT images and a 3D rendering of the labelled vessels are presented in Figure 1.

**Table 1:** Patient demographics and mean and SD of pulmonary function tests and quantitative CT data

Variable (*n* = 152 unless stated) Units are percentage unless stated	Value
Median age (years, range)	67 (38–86)
Male/female	122/30
Never/ever/current smokers	50/100/2
Alive/dead	51/101
GAP index (1/2/3) (*n* = 136)	44/73/19
FEV_1_% predicted (*n* = 138)	73.7 ± 19.4
FVC % predicted (*n* = 138)	72.7 ± 20.7
DL_CO_ % predicted (*n* = 141)	38.9 ± 12.9
K_CO_ % predicted (*n* = 141)	69.9 ± 19.0
TLC % predicted (*n* = 132)	67.2 ± 16.5
CPI (*n* = 137)	52.2 ± 11.8
Computed metrics	
Total lung volume (L)	3.9 ± 1.1
Normalized vessel volume (%)	3.6 ± 0.8
Normalized arterial volume (%;*n* = 106)	2.1 ± 0.5
Normalized venous volume (%; *n* = 106)	1.6 ± 0.4
Vessel density (vess/L)	361.5 ± 106.1
Arterial density (vess/L; *n* = 106)	198.9 ± 53.9
Venous density (vess/L; *n* = 106)	167.6 ± 48.7
Vessel tortuosity (1)	1.029 ± 0.003
Arterial tortuosity (1; *n* = 106)	1.029 ± 0.004
Venous tortuosity (1; *n* = 106)	1.029 ± 0.003
Vessel heterogeneity (1)	0.058 ± 0.006
Arterial heterogeneity (1; *n* = 106)	0.057 ± 0.007
Venous heterogeneity (1; *n* = 106)	0.058 ± 0.006

CPI, composite physiologic index; CT, computed tomography; DL_CO_, diffusion capacity for carbon monoxide; FEV_1_, forced expiratory volume in the first second; FVC, forced vital capacity; GAP, gender age physiology; K_CO_, transfer factor for carbon monoxide; TLC, total lung capacity.

### Relationships between quantitative variables

Examination of interrelationships between the quantitative vessel metrics (normalized vessel volume, vessel density, vessel tortuosity and vessel heterogeneity) demonstrated strong correlations between normalized vessel volume and vessel density, and between vessel tortuosity and vessel heterogeneity. No significant correlations were identified between normalized vessel volume and vessel tortuosity or vessel heterogeneity (Table S2, Supplementary Information).

Mean lung attenuation demonstrated a moderate negative correlation with computer‐derived total lung volume (R^2^ = 0.44, *P* < 1 × 10^−6^). Mean lung attenuation was strongly correlated with normalized vessel volume and moderately with vessel density but showed weak relationships with tortuosity and vessel heterogeneity (Table S3, Supplementary Information). Total lung volume showed significant correlations with vessel volume expressed in millilitres (R^2^ = 0.40, *P* < 1x10^−6^) and normalized to the total lung volume (R^2^ = 0.16, *P* < 1 × 10^−6^). Total lung volume was significantly associated with vessel density but correlations with vessel tortuosity and heterogeneity were weak (Table S3, Supplementary Information).

### Structure–function correlations

Relationships between quantitative CT features and functional indices are shown in Tables 2, 3, 4. Both total lung volume and mean lung attenuation measured quantitatively were correlated with FEV_1_, FVC, DL_CO_, TLC and CPI, reiterating the previously identified strong links between both measures (Table S3, Supplementary Information).

**Table 2:** Univariable analysis of various quantitative CT features with baseline FEV_1_ (top) and FVC (bottom)

Pulmonary function test	CT pattern	Number of subjects	Beta coefficient	95% CI	*P*‐value	R^2^
FEV_1_	Total lung volume (L)	136	6.54	3.87, 9.20	3 × 10^−6^	0.15
	Mean lung attenuation (HU)	136	−0.16	−0.21, −0.12	<1 × 10^−6^	0.26
	Normalized vessel volume (%)	136	−8.27	−12.00, −4.54	2 × 10^−5^	0.12
	Normalized arterial volume (%)	96	−6.09	−13.87, 1.69	0.12	0.03
	Normalized venous volume (%)	96	−8.88	−18.16, 0.40	0.06	0.04
	Vessel density (vess/L)	136	−0.05	−0.08, −0.01	0.005	0.06
	Arterial density (vess/L)	96	−0.03	−0.10, 0.04	0.35	0.01
	Venous density (vess/L)	96	−0.03	−0.11, 0.04	0.40	0.01
	Vessel heterogeneity (1)	136	−164.60	−718.64, 389.44	0.56	0.00
	Arterial heterogeneity (1)	95	217.28	−321.16, 755.95	0.43	0.01
	Venous heterogeneity (1)	95	58.42	−550.02, 666.86	0.85	0.00
FVC	Total lung volume (L)	136	10.59	8.08, 13.11	<1 × 10^−6^	0.34
	Mean lung attenuation (HU)	136	−0.24	−0.28, −0.19	<1 × 10^−6^	0.47
	Normalized vessel volume (%)	136	−13.14	−16.77, −9.51	<1 × 10^−6^	0.27
	Normalized arterial volume (%)	96	−16.18	−23.69, −8.68	5 × 10^−5^	0.16
	Normalized venous volume (%)	96	−20.29	−29.22, −11.36	2 × 10^−5^	0.18
	Vessel density (vess/L)	136	−0.08	−0.11, −0.05	1 × 10^−6^	0.16
	Arterial density (vess/L)	96	−0.12	−0.19 ‐0.05	0.001	0.11
	Venous density (vess/L)	96	−0.12	−0.20, −0.05	0.003	0.10
	Vessel heterogeneity (1)	136	−471.88	−1060.19, 116.44	0.12	0.02
	Arterial heterogeneity (1)	96	−88.94	−650.98, 473.11	0.75	0.00
	Venous heterogeneity (1)	96	−396.40	−1024.20, 232.07	0.21	0.02

CT, computed tomography; FEV_1_, forced expiratory volume in the first second; FVC, forced vital capacity; HU, Hounsfield Unit.

**Table 3:** Univariable analysis of various quantitative CT features with baseline DL_CO_ (top) and K_CO_ (bottom)

Pulmonary function test	CT pattern	Number of subjects	Beta coefficient	95% CI	*P*‐value	R^2^
DL_CO_	Total lung volume (L)	139	3.46	1.68, 5.25	0.0002	0.10
	Mean lung attenuation (HU)	139	−0.11	−0.14, −0.08	<1 × 10^−6^	0.25
	Normalized vessel volume (%)	139	−5.43	−7.90, −2.96	3 × 10^−5^	0.12
	Normalized arterial volume (%)	96	−9.48	−14.74, −4.23	0.001	0.12
	Normalized venous volume (%)	96	−8.06	−14.65, −1.47	0.02	0.06
	Vessel density (vess/L)	139	−0.05	−0.07, −0.03	<1 × 10^−6^	0.17
	Arterial density (vess/L)	96	−0.08	−0.13, −0.03	0.001	0.11
	Venous density (vess/L)	96	−0.08	−0.13, −0.02	0.008	0.07
	Vessel heterogeneity (1)	139	−397.93	−755.89, −39.97	0.03	0.03
	Arterial heterogeneity (1)	96	−221.35	−599.12, 156.42	0.25	0.01
	Venous heterogeneity (1)	96	−203.23	−629.48, 223.02	0.35	0.01
K_CO_	Total lung volume (L)	139	3.85	−6.53, −1.17	0.005	0.06
	Mean lung attenuation (HU)	139	0.02	−0.03, 0.07	0.47	0.00
	Normalized vessel volume (%)	139	1.99	−1.86, 5.85	0.31	0.01
	Normalized arterial volume (%)	96	0.95	−7.41, 9.31	0.82	0.01
	Normalized venous volume (%)	96	5.74	−4.33, 15.82	0.26	0.01
	Vessel density (vess/L)	139	−0.02	−0.05, 0.01	0.24	0.01
	Arterial density (vess/L)	96	−0.00	−0.08, 0.07	0.96	0.00
	Venous density (vess/L)	96	−0.00	−0.08, 0.09	0.94	0.00
	Vessel heterogeneity (1)	139	−136.95	−671.13, 397.24	0.61	0.00
	Arterial heterogeneity (1)	96	−125.38	−692.96, 442.20	0.66	0.00
	Venous heterogeneity (1)	96	219.99	−417.98, 857.96	0.69	0.01

CT, computed tomography; DL_CO_, diffusion capacity for carbon monoxide; HU, Hounsfield Unit; K_CO_, transfer factor for carbon monoxide.

**Table 4:** Univariable analysis of various quantitative CT features with baseline TLC (top) and the CPI (bottom)

Pulmonary function test	CT pattern	Number of subjects	Beta coefficient	95% CI	*P*‐value	R^2^
TLC	Total lung volume (L)	131	9.69	7.89, 11.49	<1 × 10^−6^	0.46
	Mean lung attenuation (HU)	131	−0.21	−0.024, −0.18	<1 × 10^−6^	0.61
	Normalized vessel volume (%)	131	−13.25	−15.77, −10.72	<1 × 10^−6^	0.45
	Normalized arterial volume (%)	90	−22.73	−28.31, −17.15	<1 × 10^−6^	0.42
	Normalized venous volume (%)	90	−25.11	−31.94, −18.29	<1 × 10^−6^	0.38
	Vessel density (vess/L)	131	−0.09	−0.11, −0.07	<1 × 10^−6^	0.31
	Arterial density (vess/L)	90	−0.17	−0.22, −0.12	<1 × 10^−6^	0.31
	Venous density (vess/L)	90	−0.17	−0.23, −0.10	6 × 10^−6^	0.24
	Vessel heterogeneity (1)	131	−485.65	−968.88, −2.42	0.049	0.03
	Arterial heterogeneity (1)	90	−235.98	−739.71, 267.75	0.35	0.01
	Venous heterogeneity (1)	90	−539.40	−1093.49, 14.69	0.06	0.04
CPI	Total lung volume (L)	136	−5.49	−6.99, −4.00	<1 × 10^−6^	0.28
	Mean lung attenuation (HU)	136	0.14	0.12, 0.16	<1 × 10^−6^	0.50
	Normalized vessel volume (%)	136	7.66	5.58, 9.74	<1 × 10^−6^	0.28
	Normalized arterial volume (%)	95	12.51	8.23, 16.80	1 × 10^−6^	0.26
	Normalized venous volume (%)	95	12.75	7.29, 18.22	1 × 10^−5^	0.19
	Vessel density (vess/L)	136	0.06	0.04, 0.08	<1 × 10^−6^	0.27
	Arterial density (vess/L)	95	0.10	0.07, 0.14	1 × 10^−5^	0.23
	Venous density (vess/L)	95	0.10	0.06, 0.15	5 × 10^−5^	0.16
	Vessel heterogeneity (1)	136	453.44	124.82, 782.07	0.007	0.05
	Arterial heterogeneity (1)	95	267.12	−67.44, 601.68	0.12	0.03
	Venous heterogeneity (1)	95	360.57	−13.48, 735.36	0.06	0.04

CPI, composite physiologic index; CT, computed tomography; HU, Hounsfield Unit; TLC, total lung capacity.

Of the vessel metrics, normalized vessel volume demonstrated the strongest linkages with FEV_1_, FVC, TLC and CPI (Tables 2, 4). Vessel density was weakly but significantly linked with these functional parameters. Vessel tortuosity and vessel heterogeneity were not strongly linked with any functional indices. As the correlations were similarly weak for vessel tortuosity and heterogeneity, only results for the latter are shown in Tables 2, 3, 4. None of the quantitative vessel metrics was significantly linked to K_CO_. Normalized vessel volume (R^2^ = 0.12, *P* = 3 × 10^−5^) and vessel density (R^2^ = 0.16, *P* = 1 × 10^−6^) showed weak links with DL_CO_, but vessel tortuosity or heterogeneity did not. Therefore, the readouts of macrovascular morphology showed at best only weak correlations with the two functional parameters that may contain information on small vessel disease.

When vessel metrics were subdivided according to zonal location in the z‐axis, lower zone metrics correlated weakly with FVC, DL_CO_ and CPI compared to upper zone or middle zone metrics. Middle zone metrics demonstrated slightly stronger functional correlations than upper zone metrics for DL_CO_ but were equivalent for CPI and weaker for FVC (Table S4, Supplementary Information).

On multivariable linear regression analyses, in models adjusted for patient age, male gender, smoking status and CT slice thickness (0.7 or 1.0 mm), normalized vessel volume and vessel heterogeneity were independently linked to DL_CO_, TLC and CPI (Table 5). Vessel heterogeneity in the middle zones showed stronger independent links with DL_CO_ and CPI than upper or lower zone heterogeneity (Table S5, Supplementary Information). Vessel density was not examined in the multivariable models due to strong collinearity with normalized vessel volume. Vessel tortuosity was not correlated with any functional indices in the multivariable analysis.

**Table 5:** Multivariable linear regression analyses demonstrating relationships between pulmonary functional indices and total vessel volume and vessel heterogeneity metrics

Dependent variable	Independent variable	Beta coefficient	95% CI	*P*‐value	R^2^
FEV_1_	Normalized vessel volume (%)	−6.78	−10.68, −2.87	0.001	0.21
	Vessel heterogeneity (1)	−126.45	−518.05, 770.95	0.70	
FVC	Normalized vessel volume (%)	−11.61	−15.40, −7.82	<1 × 10^−6^	0.35
	Vessel heterogeneity (1)	−214.05	−839.19, 411.08	0.50	
DL_CO_	Normalized vessel volume (%)	−5.58	−8.16, −3.501	4 × 10^−5^	0.21
	Vessel heterogeneity (1)	−680.13	−1103.32, −256.93	0.002	
K_CO_	Normalized vessel volume (%)	1.13	−2.83, 5.08	0.57	0.15
	Vessel heterogeneity (1)	−548.55	−1198.28, 101.18	0.10	
TLC	Normalized vessel volume (%)	−12.63	−15.28, −9.99	<1 × 10^−6^	0.51
	Vessel heterogeneity (1)	−528.32	−968.25, −88.39	0.01	
CPI	Normalized vessel volume (%)	7.46	5.28, 9.65	<1 × 10^−6^	0.34
	Vessel heterogeneity (1)	558.28	231.26, 945.30	0.001	

All models were adjusted for patient age, male gender, smoking status (never vs ever) and CT slice thickness (0.7 vs 1.0 mm).

CPI, composite physiologic index; CT, computed tomography; DL_CO_, diffusion capacity for carbon monoxide; FEV_1_, forced expiratory volume in the first second; FVC, forced vital capacity; K_CO_, transfer factor for carbon monoxide; TLC, total lung capacity.

### Artery and vein separation

Correlations were strong between normalized arterial and venous volume (R^2^ = 0.70, *P* < 1 × 10^−6^), arterial and venous density (R^2^ = 0.83, *P* < 1 × 10^−6^), arterial and venous tortuosity (R^2^ = 0.68, *P* < 1 × 10^−6^) and arterial and venous heterogeneity (R^2^ = 0.54, *P* < 1 × 10^−6^). Strong links between normalized vessel volume and vessel density and between vessel tortuosity and vessel heterogeneity were maintained when arteries and veins were examined separately (Table S2, Supplementary Information). All functional linkages for normalized vessel volume and density were maintained, although weakly, when separately examined in arteries and veins (Tables 2, 3, 4).

## DISCUSSION

Our study has used a novel vessel quantitation tool to confirm previous observations of correlations between the normalized volume of pulmonary vessels and lung function indices in IPF patients. The number of vessels per lung volume (vessel density), a new quantitative vessel parameter, was strongly correlated with the normalized vessel volume and also correlated to measures of lung function. The vessel heterogeneity (distribution width of tortuosity) was independently linked with DL_CO_, TLC and CPI. No vessel metric was significantly correlated with K_CO_ whilst there were weak linkages with DL_CO_, suggesting that macrovascular morphology is poorly linked to microvascular disease. Vessel metrics distant to areas of fibrosis demonstrated stronger functional correlations than metrics in the lower lung zones. Separation of arteries and veins demonstrated no additional functional information beyond that explained by the whole vasculature.

The importance of the pulmonary vessels as prognostic indices in IPF has primarily been considered in relation to pulmonary hypertension. The main pulmonary artery diameter,^16^ the pulmonary artery/aorta ratio^17^ and quantitatively derived vessel tortuosity^15^ have been shown to predict the likelihood of pulmonary hypertension and of exacerbations in COPD.^18^ However, quantitation of the combined pulmonary arteries and veins (excluding hilar vessels) by a computer tool, CALIPER, linked strongly to functional indices^7^ and mortality,^8^ but only weakly with indirect measures of pulmonary hypertension. The current analysis shows that further readouts of vessel morphology correlate with lung function parameters and may therefore also be of relevance as prognostic markers in IPF.

We identified relatively stronger inverse relationships between lung function indices and vessel volume in the upper/middle zones compared to the lower lung zones. These results are in line with previous reports of stronger links between FVC decline and mortality in baseline upper/middle zone VRS when compared to lower zone VRS in patients with IPF.^9^ MRI studies have demonstrated that fibrotic tissues show delayed contrast enhancement when compared to morphologically normal appearing lung in lung fibrosis patients.^19^, ^20^ A local increase in pulmonary vascular resistance in fibrotic areas may reduce regional pulmonary blood flow due to both hypoxic vasoconstriction and proliferative remodelling caused by inflammation. This may cause increased pulmonary arterial pressure and increased vessel size in unaffected vessels in regions of normal lung parenchyma which may therefore act as a surrogate marker of both pulmonary hypertension and the extent of interstitial disease.

In addition to vessel tortuosity, we evaluated a new metric of vessel heterogeneity that captured variations in vessel tortuosity which would be expected to differ between regions of fibrosis and normal lung parenchyma. Examining vessel heterogeneity was motivated by the histopathological description of IPF as spatially and temporally heterogeneous disease.^21^ The relationship of vessel heterogeneity with lung function indices was strongest in the lung mid‐zones which contain the largest mix of normal and fibrotic tissue in IPF lungs and therefore the widest range of vascular morphology. Further work is necessary to determine how vessel tortuosity is linked with the development and progression of fibrosis and how this might influence local changes in perfusion.

Linkages were identified between quantitatively derived total lung volume and mean lung attenuation reflecting the increase in parenchymal collagen deposition and functional airspace loss as well as the concomitant shrinkage of the functional lung volume as a result of lung fibrosis. Mean lung attenuation, which captures a global picture of fibrosis extent/severity in the lung, was strongly correlated with normalized vessel volume and vessel density. The association of normalized vessel volume and vessel density with overall fibrosis extent was also captured in the strong links for both variables with CPI which reflects the morphological extent of lung fibrosis on a CT scan.^11^ The observed linkages all go to confirm previous reports describing marked associations between increases in normalized vessel volume with increased total extent of ILD.^7^ Intriguingly, vessel heterogeneity did not demonstrate linkages with mean lung attenuation, total lung volume or total vessel volume, yet was independently linked to TLC and CPI, suggesting that vessel heterogeneity might reflect an independent facet of lung damage.

Functional correlations of quantitative artery and vein metrics were similar or weaker than total vessel metrics. The challenges associated with performing accurate artery/vein separation are considerable and our findings suggest that quantifying total lung vessels might yet be a more pragmatic approach to evaluating functionally important measures in IPF.

There were limitations to the current study. Patients excluded from our analysis had significantly more severe disease than study patients, which may have introduced bias into our analysis. However, the mean DL_CO_ in our study cohort (39.1%) was similar to the mean DL_CO_ (43.7%) in the landmark Pirfenidone trial,^22^ indicating that our study population constituted an acceptable degree of baseline disease severity. We had no right heart catheterization data to examine links between quantitative vessel metrics and measures of pulmonary hypertension. Data sets of patients with concomitant CT imaging and right heart catheterization are likely to be encumbered by selection bias of their own and are rare in practice. Yet, such analyses remain a goal for future work.

In conclusion, we have shown that vessel metrics quantified by a novel algorithm for lung vessel analysis link strongly to lung function in IPF validating previously presented results using a different vessel quantification tool. Quantitative vessel metrics demonstrated weak overall linkages with DL_CO_ and K_CO_, suggesting that metrics of macrovessels do not reflect functional microvasculopathy. Whilst total upper zone vessel metrics better predicted functional indices than lower zone metrics, separation of vessel metrics into pulmonary artery and vein subdivisions did not enhance functional correlations when compared to equivalent total vessel scores.

## Disclosure statement

J.J. reports personal fees from Boehringer Ingelheim unrelated to the submitted work. A.D. reports personal fees from Boehringer Ingelheim and Roche unrelated to the submitted work. H.O. reports personal fees from participating in advisory boards and speaking at symposia from Actelion, Astra Zeneca, Bayer, Bellerophon, Boehringer Ingelheim, Chiesi, GSK, Menarini, MSD, Novartis and Pfizer, and received research grants from Actelion, Boehringer Ingelheim, Roche and Inventiva unrelated to the submitted work. A.U.W. receives personal fees for participating in advisory boards and speaking at symposia from Boehringer Ingelheim, Intermune, Roche and Bayer, and for participating in advisory boards from Gilead, MSD and speaker fees from Chiesi.

**Abbreviations**
CPI: composite physiologic index
CT: computed tomography
DL_CO_: diffusion capacity for carbon monoxide
FEV_1_: forced expiratory volume in the first second
FVC: forced vital capacity
HU: Hounsfield Unit
ILD: interstitial lung disease
IPF: idiopathic pulmonary fibrosis
K_CO_: transfer factor for carbon monoxide
MRI: magnetic resonance imaging
PFT: pulmonary function test
TLC: total lung capacity
VRS: vessel‐related structure

## Supporting information


**Appendix S1** Additional methods.
**Table S1** Group differences between patients included and excluded from the study population.
**Table S2** Relationships between quantitative vessel metrics.
**Table S3** Relationships between total lung volume and mean lung attenuation quantified by computer analysis, with computer‐derived vessel metrics.
**Table S4** Relationships between pulmonary functional indices and total vessel metrics subdivided according to three equal sized lung zones.
**Table S5** Multivariable linear regression relationships between pulmonary functional indices and zonal total vessel volume and vessel heterogeneity metrics.
