# Supplementary material for: Quantitative CT‐derived vessel metrics in idiopathic pulmonary fibrosis: A structure–function study
Source: Respirology. 2019 Feb 20;24(5):445–52. doi: 10.1111/resp.13485 (PMC6519024; doi:10.1111/resp.13485)
Supplement: Supplementary file 1 — Appendix S1 Additional methods. Table S1 Group differences between patients included and excluded from the study population. Table S2 Relationships between quantitative vessel metrics. Table S3 Relationships between total lung volume and mean lung attenuation quantified by computer analysis, with computer‐derived vessel metrics. Table S4 Relationships between pulmonary functional indices and total vessel metrics subdivided according to three equal sized lung zones. Table S5 Multivariable linear regression relationships between pulmonary functional indices and zonal total vessel volume and vessel heterogeneity metrics. [file RESP-24-445-s001.docx]

**SUPPLEMENTARY INFORMATION**

**Quantitative CT-derived vessel metrics in idiopathic pulmonary fibrosis: A structure function study**

Joseph Jacob^1,2*^, Michael Pienn^3*^, Christian Payer^4^, Martin Urschler^4,5^, Maria Kokosi^6^, Anand Devaraj^7^, Athol U. Wells^6^, Horst Olschewski^3,8^.

^1^Department of Respiratory Medicine, University College London, UK.

^2^Centre for Medical Image Computing, University College London, UK.

^3^Ludwig Boltzmann Institute for Lung Vascular Research, Graz, Austria.

^4^Institute of Computer Graphics & Vision, Graz University of Technology, Graz, Austria.

^5^Ludwig Boltzmann Institute for Clinical-Forensic Imaging, Graz, Austria.

^6^Interstitial Lung Disease Unit, Royal Brompton Hospital, London, UK.

^7^Department of Radiology, Royal Brompton Hospital, London, UK.

^8^Division of Pulmonology, Department of Internal Medicine, Medical University of Graz, Graz, Austria.

**Appendix S1-** *Additional Methods*

**CT Protocols**

The CT scans were obtained using a 64-slice multiple detector CT scanner (Somatom Sensation 64, Siemens, Erlangen, Germany) or a 4-slice multiple detector CT scanner (Siemens Volume Zoom, Siemens, Erlangen, Germany). All patients were scanned from lung apices to bases, supine, at full inspiration using a peak voltage of 120kVp with tube current modulation (range 30-140 mA). The scans were reconstructed using a high spatial frequency, B70 kernel (Siemens, Munich, Germany) with 0.7 or 1.0mm slice thicknesses. Images were viewed at window settings optimized for the assessment of the lung parenchyma (width 1500 HU; level -500 HU).

**Pulmonary function tests:**

Pulmonary function tests were analyzed if they were performed within 3 months of the corresponding CT scan according to established protocols(1). They included spirometry (Jaeger Master screen PFT, Carefusion Ltd., Warwick, UK), plethysmographic lung volumes (Jaeger Master screen Body, Carefusion Ltd., Warwick, UK), and diffusion capacity for carbon monoxide (DLco) (Jaeger Master screen PFT, Carefusion Ltd., Warwick. UK). The parameters assessed were forced expiratory volume in one second (FEV1), forced vital capacity (FVC), total lung capacity (TLC), transfer coefficient of the lung for carbon monoxide (Kco) and single breath carbon monoxide diffusing capacity corrected for hemoglobin concentration (DLco). The composite physiologic index (CPI) was calculated using the formula: 91.0 - (0.65 x % predicted DLco) - (0.53 x % predicted FVC) + (0.34 x % predicted FEV1)(2).

**Table S1-** Group differences between patients included and excluded from the study population

| **Variable** | **Patients included**  **in study** | **Patients excluded**  **from study** | **P value** |
| --- | --- | --- | --- |
| Median age (years; range) | 67 (38-86) | 70 (48-85) | 0.14^*^ |
| Gender (M/F) | 122/30 | 17/7 | 0.29^‡^ |
| Smoking (never vs ever) | 50/102 | 11/13 | 0.22^‡^ |
| Mean FVC (% predicted) | 72.7±20.7 | 53.5±15.8 | 0.00002^§^ |
| Mean DLco (% predicted) | 38.9±12.9 | 28.1±10.3 | 0.0001^§^ |

Supplementary Table 1. Group differences between patients included in the study (n=152) and patients excluded from the study (n=24). Patients were excluded for three reasons: a) the CT image could not be segmented, b) >10% of vessels were not segmented, and

c) >10% of the structures classified as vessels were non-vascular fibrotic artefacts. *=Mann-Whitney U test, ‡=Chi-squared test, §=Student t-test.

**Table S2-** Relationships between quantitative vessel metrics

| **Dependent variable** | | **Independent variable** | **Beta Coefficient** | **95% Confidence Interval** | **P value** | **R^2^** |
| --- | --- | --- | --- | --- | --- | --- |
| Normalized vessel volume (%) | Normalized arterial volume (%) | | 1.70 | 1.61, 1.79 | <1x10^-6^ | 0.93 |
|  | Normalized venous volume (%) | | 1.98 | 1.85, 2.10 | <1x10^-6^ | 0.90 |
| Vessel density (vess/L) | Normalized vessel volume (%) | | 94.51 | 79.93, 109.09 | <1x10^-6^ | 0.52 |
|  | Normalized arterial volume (%) | | 145.89 | 114.88, 176.90 | <1x10^-6^ | 0.46 |
|  | Normalized venous volume (%) | | 181.85 | 146.98, 216.72 | <1x10^-6^ | 0.51 |
| Vessel tortuosity (1) | Normalized vessel volume (%) | | -0.00 | -0.00, 0.00 | 0.007 | 0.05 |
|  | Normalized arterial volume (%) | | -0.00 | -0.00, 0.00 | 0.15 | 0.02 |
|  | Normalized venous volume (%) | | -0.00 | -0.00, 0.00 | 0.17 | 0.02 |
| Vessel heterogeneity (%) | Normalized vessel volume (%) | | 0.00 | -0.00, 0.00 | 0.91 | 0.01 |
|  | Normalized arterial volume (%) | | 0.00 | -0.00, 0.00 | 0.46 | 0.01 |
|  | Normalized venous volume (%) | | 0.00 | -0.00, 0.00 | 0.49 | 0.01 |
| Normalized vessel volume (%) | Arterial density (vess/L) | | 0.01 | 0.01, 0.01 | <1x10^-6^ | 0.54 |
|  | Venous density (vess/L) | | 0.01 | 0.01, 0.01 | <1x10^-6^ | 0.46 |
| Vessel density (vess/L) | Arterial density (vess/L) | | 1.82 | 1.75, 1.90 | <1x10^-6^ | 0.96 |
|  | Venous density (vess/L) | | 2.01 | 1.92, 2.10 | <1x10^-6^ | 0.95 |
| Vessel tortuosity (1) | Vessel density (vess/L) | | -2x10^-5^ | -2x10^-5^, -4x10^-6^ | 0.0004 | 0.08 |
|  | Arterial density (vess/L) | | -1x10^-5^ | -3x10^-5^, -1x10^-6^ | 0.03 | 0.04 |
|  | Venous density (vess/L) | | -2x10^-5^ | -3x10^-5^, -2x10^-6^ | 0.02 | 0.05 |
| Vessel heterogeneity (%) | Vessel density (vess/L) | | 6x10^-6^ | -4x10^-6^, 2x10^-5^ | 0.23 | 0.01 |
|  | Arterial density (vess/L) | | 2x10^-5^ | -4x10^-6^, 4x10^-5^ | 0.12 | 0.02 |
|  | Venous density (vess/L) | | 2x10^-5^ | -5x10^-6^, 4x10^-5^ | 0.12 | 0.02 |
| Normalized vessel volume (%) | Arterial tortuosity (1) | | -34.99 | -77.69, 7.70 | 0.11 | 0.03 |
|  | Venous tortuosity (1) | | -28.32 | -74.40, 17.7 | 0.23 | 0.01 |
| Vessel density (vess/L) | Arterial tortuosity (1) | | -6578.16 | -11729.67, -1426.65 | 0.01 | 0.06 |
|  | Venous tortuosity (1) | | -4948.55 | -10565.01, 667.91 | 0.08 | 0.03 |
| Vessel tortuosity (1) | Arterial tortuosity (1) | | 0.89 | 0.84, 0.94 | <1x10^-6^ | 0.92 |
|  | Venous tortuosity (1) | | 0.94 | 0.87, 1.00 | <1x10^-6^ | 0.89 |
| Vessel heterogeneity (%) | Vessel tortuosity (1) | | 1.35 | 1.17, 1.53 | <1x10^-6^ | 0.59 |
|  | Arterial tortuosity (1) | | 1.26 | 1.04, 1.48 | <1x10^-6^ | 0.56 |
|  | Venous tortuosity (1) | | 1.35 | 1.12, 1.59 | <1x10^-6^ | 0.55 |
| Normalized vessel volume (%) | Arterial heterogeneity (1) | | 5.01 | -17.65, 27.66 | 0.66 | 0.00 |
|  | Venous heterogeneity (1) | | 10.07 | -14.52, 34.66 | 0.42 | 0.01 |
| Vessel density (vess/L) | Arterial heterogeneity (1) | | 1821.15 | -940.27, 4582.56 | 0.19 | 0.02 |
|  | Venous heterogeneity (1) | | 2585.19 | -401.56, 5571.94 | 0.09 | 0.03 |
| Vessel tortuosity (1) | Arterial heterogeneity (1) | | 0.36 | 0.30, 0.42 | <1x10^-6^ | 0.55 |
|  | Venous heterogeneity (1) | | 0.37 | 0.30, 0.44 | <1x10^-6^ | 0.49 |
| Vessel heterogeneity (%) | Arterial heterogeneity (1) | | 0.84 | 0.79, 0.90 | <1x10^-6^ | 0.90 |
|  | Venous heterogeneity (1) | | 0.88 | 0.80, 0.96 | <1x10^-6^ | 0.82 |

Supplementary Table 2. Univariable linear regression analyses demonstrating relationships between various quantitative vessel metrics described for total vessels, and separately for arteries and veins. All artery and vein analyses were performed in 106 patients; all other analyses were performed in 152 patients.

**Table S3-** Relationships between total lung volume and mean lung attenuation quantified by computer analysis, with computer-derived vessel metrics

| **Computed metric** | | **CT pattern** | **Beta Coefficient** | **95% Confidence Interval** | **P value** | **R^2^** |
| --- | --- | --- | --- | --- | --- | --- |
| Total lung volume (L, quantitative) | Mean lung attenuation (HU) | | -0.01 | -0.02, -0.01 | <1x10^-6^ | 0.44 |
|  | Normalized vessel volume (%) | | -0.55 | -0.76, -0.35 | <1x10^-6^ | 0.16 |
|  | Normalized arterial volume (%) | | -0.89 | -1.33, -0.44 | 0.0001 | 0.13 |
|  | Normalized venous volume (%) | | -1.24 | -1.75, -0.73 | 1x10^-6^ | 0.18 |
|  | Vessel density (vess/L) | | -0.00 | -0.01, -0.00 | 1x10^-6^ | 0.15 |
|  | Arterial density (vess/L) | | -0.01 | -0.01, -0.01 | 3x10^-6^ | 0.19 |
|  | Venous density (vess/L) | | -0.01 | -0.01, -0.01 | 1x10^-5^ | 0.17 |
|  | Vessel heterogeneity (1) | | -54.29 | -83.48, -25.11 | 0.0004 | 0.08 |
|  | Arterial heterogeneity (1) | | -52.63 | -82.59, -22.68 | 0.001 | 0.11 |
|  | Venous heterogeneity (1) | | -52.65 | -85.52, -19.77 | 0.002 | 0.09 |
| Mean lung attenuation (HU) | Normalized vessel volume (%) | | 54.36 | 46.40, 62.32 | <1x10^-6^ | 0.55 |
|  | Normalized arterial volume (%) | | 94.92 | 76.38, 113.46 | <1x10^-6^ | 0.50 |
|  | Normalized venous volume (%) | | 116.21 | 95.11, 137.30 | <1x10^-6^ | 0.53 |
|  | Vessel density (vess/L) | | 0.29 | 0.21, 0.37 | <1x10^-6^ | 0.26 |
|  | Arterial density (vess/L) | | 0.66 | 0.47, 0.84 | <1x10^-6^ | 0.32 |
|  | Venous density (vess/L) | | 0.66 | 0.45, 0.88 | <1x10^-6^ | 0.27 |
|  | Vessel heterogeneity (1) | | 2441.64 | 885.17, 3998.11 | 0.002 | 0.06 |
|  | Arterial heterogeneity (1) | | 2547.00 | 886.23, 4207.76 | 0.003 | 0.08 |
|  | Venous heterogeneity (1) | | 2598.63 | 782.23, 4415.04 | 0.005 | 0.07 |

Supplementary Table 3. Univariable linear regression analyses demonstrating relationships between total lung volume (top) and mean lung attenuation (bottom) quantified by computer analysis, with various expressions of total vessel, artery and vein quantitation using computer analysis. All artery and vein analyses were performed in 106 patients; all other analyses were performed in 152 patients.

**Table S4-** Relationships between pulmonary functional indices and total vessel metrics subdivided according to three equal sized lung zones

| **Dependent variable** | **Independent variable** | **Beta Coefficient** | **95% Confidence Interval** | **P value** | **R^2^** |
| --- | --- | --- | --- | --- | --- |
| FVC | UZ Normalized vessel volume (%) | -12.12 | -15.20, -9.04 | <1x10^-6^ | 0.31 |
|  | MZ Normalized vessel volume (%) | -12.49 | -15.96, -9.02 | <1x10^-6^ | 0.27 |
|  | LZ Normalized vessel volume (%) | -7.67 | -11.03, -4.32 | 1x10^-5^ | 0.13 |
|  | UZ Vessel density (vess/L) | -0.09 | -0.11, -0.06 | <1x10^-6^ | 0.25 |
|  | MZ Vessel density (vess/L) | -0.08 | -0.12, -0.05 | 1x10^-6^ | 0.16 |
|  | LZ Vessel density (vess/L) | -0.04 | -0.07, -0.01 | 0.01 | 0.05 |
|  | UZ Vessel heterogeneity (1) | -457.56 | -953.46, 38.35 | 0.07 | 0.02 |
|  | MZ Vessel heterogeneity (1) | -541.44 | -1103.42, 20.53 | 0.06 | 0.03 |
|  | LZ Vessel heterogeneity (1) | -50.71 | -532.35, 430.92 | 0.84 | 0.00 |
| DLco | UZ Normalized vessel volume (%) | -4.47 | -6.64, -2.29 | 8x10^-5^ | 0.11 |
|  | MZ Normalized vessel volume (%) | -5.59 | -7.92, -3.26 | 5x10^-6^ | 0.14 |
|  | LZ Normalized vessel volume (%) | -3.51 | -5.62, -1.40 | 0.001 | 0.07 |
|  | UZ Vessel density (vess/L) | -0.04 | -0.06, -0.03 | 2x10^-6^ | 0.15 |
|  | MZ Vessel density (vess/L) | -0.06 | -0.07, -0.04 | <1x10^-6^ | 0.20 |
|  | LZ Vessel density (vess/L) | -0.03 | -0.05, -0.02 | 1x10^-4^ | 0.10 |
|  | UZ Vessel heterogeneity (1) | -284.24 | -588.08, 19.60 | 0.07 | 0.02 |
|  | MZ Vessel heterogeneity (1) | -453.82 | 792.54, -115.09 | 0.009 | 0.05 |
|  | LZ Vessel heterogeneity (1) | -161.56 | -456.71, 133.59 | 0.28 | 0.01 |
| CPI | UZ Normalized vessel volume (%) | 6.69 | 4.88, 8.49 | <1x10^-6^ | 0.29 |
|  | MZ Normalized vessel volume (%) | 7.46 | 5.49, 9.43 | <1x10^-6^ | 0.29 |
|  | LZ Normalized vessel volume (%) | 4.63 | 2.72, 6.53 | 4x10^-6^ | 0.15 |
|  | UZ Vessel density (vess/L) | 0.06 | 0.04, 0.07 | <1x10^-6^ | 0.29 |
|  | MZ Vessel density (vess/L) | 0.06 | 0.05, 0.08 | <1x10^-6^ | 0.29 |
|  | LZ Vessel density (vess/L) | 0.04 | 0.02, 0.05 | 3x10^-5^ | 0.12 |
|  | UZ Vessel heterogeneity (1) | 398.71 | 121.24, 676.18 | 0.005 | 0.06 |
|  | MZ Vessel heterogeneity (1) | 494.84 | 183.06, 807.63 | 0.002 | 0.07 |
|  | LZ Vessel heterogeneity (1) | 174.57 | -97.82, 446.97 | 0.21 | 0.01 |

Supplementary Table 4. Univariable linear regression analyses demonstrating relationships between pulmonary functional indices and total vessel metrics subdivided according to three equal sized zones of the lungs (upper zone=UZ, middle zone=MZ, lower zone=LZ). FVC=forced vital capacity, DLco=diffusing capacity for carbon monoxide, CPI=composite physiologic index.

**Table S5-** Multivariable linear regression relationships between pulmonary functional indices and zonal total vessel volume and vessel heterogeneity metrics.

| **Functional Index** | **Dependent variable** | | **Independent variable** | **Beta Coefficient** | **95% Confidence Interval** | **P value** | **R^2^** |
| --- | --- | --- | --- | --- | --- | --- | --- |
| DLco | Upper Zone | Normalized vessel volume (%) | | -4.32 | -6.60, -2.05 | 0.0003 | 0.16 |
|  |  | Vessel heterogeneity (1) | | -375.48 | -725.54, -25.42 | 0.04 |  |
| DLco | Middle Zone | Normalized vessel volume (%) | | -5.06 | -7.52, -2.60 | <1x10^-6^ | 0.21 |
|  |  | Vessel heterogeneity (1) | | -545.23 | -918.26, -172.20 | 0.004 |  |
| DLco | Lower Zone | Normalized vessel volume (%) | | -4.16 | -6.45, -1.86 | 0.0005 | 0.13 |
|  |  | Vessel heterogeneity (1) | | -330.81 | -661.17, -0.44 | 0.05 |  |
| CPI | Upper Zone | Normalized vessel volume (%) | | 6.32 | 4.43, 8.21 | <1x10^-6^ | 0.33 |
|  |  | Vessel heterogeneity (1) | | 428.24 | 138.83, 717.64 | 0.004 |  |
| CPI | Middle Zone | Normalized vessel volume (%) | | 6.93 | 4.82, 9.04 | <1x10^-6^ | 0.33 |
|  |  | Vessel heterogeneity (1) | | 409.63 | 91.30, 727.95 | 0.01 |  |
| CPI | Lower Zone | Normalized vessel volume (%) | | 4.85 | 2.79, 6.91 | 8x10^-6^ | 0.18 |
|  |  | Vessel heterogeneity (1) | | 282.26 | -14.11, 578.62 | 0.06 |  |

Supplementary Table 5. Multivariable linear regression analyses demonstrating relationships between pulmonary functional indices (DLco and CPI) and zonal total vessel volume and vessel heterogeneity metrics. All models were adjusted for patient age, male gender, smoking status (never vs. ever) and CT slice thickness (0.7mm versus 1.0mm). Analyses were separately performed for quantitative vascular metrics in the upper, middle and lower zones. DLco=diffusing capacity for carbon monoxide, CPI=composite physiologic index.

**REFERENCES**

1. Quanjer PH. Standardized lung function testing. Eur Respir J - Suppl. 1993;6:1-100.

2. Wells AU, Desai SR, Rubens MB, Goh NS, Cramer D, Nicholson AG, Colby TV, Du Bois RM, Hansell DM. Idiopathic pulmonary fibrosis: a composite physiologic index derived from disease extent observed by computed tomography. Am J Respir Crit Care Med. 2003;167:962-9.
